# The Emerging Role of Triggering Receptor Expressed on Myeloid Cells 2 as a Target for Immunomodulation in Ischemic Stroke

**DOI:** 10.3389/fimmu.2019.01668

**Published:** 2019-07-17

**Authors:** Pascal Gervois, Ivo Lambrichts

**Affiliations:** Department of Morphology, Biomedical Research Institute, Hasselt University, Diepenbeek, Belgium

**Keywords:** ischemic stroke, microglia, TREM2, immunomodulation, phagocytosis

## Abstract

Stroke is the second most common cause of death and permanent disability. It is characterized by loss of neural tissue in which inflammation plays a crucial role in both the acute contribution to ischemic damage as in the late-stage impact on post-ischemic tissue regeneration. Microglia play a key role in the inflammatory stroke microenvironment as they can adapt a disease-promoting pro-inflammatory- or pro-regenerative phenotype thereby contributing to the exacerbation or alleviation of ischemic damage, respectively. Triggering receptor expressed on myeloid cells 2 (TREM2) is a cell surface receptor which in the central nervous system is mainly expressed on microglia. This receptor has been shown to play an essential role in microglial phagocytosis and function but its contribution in stroke pathobiology remains unclear. TREM2 was shown to be activated by nucleotides and lipid mediators, key factors that are secreted in the extracellular stroke environment by apoptotic neurons and cell/myelin debris. These factors in turn stimulate TREM2 signaling which mediates microglial migration toward- and phagocytosis of myelin debris and apoptotic cells. Moreover, microglial TREM2 appears to counteract the toll-like receptor response, thereby decreasing the production of pro-inflammatory cytokines. Finally, TREM2 is involved in microglial migration, survival, and is suggested to directly stimulate pro-regenerative phenotype shift. Therefore, this receptor is an attractive target for microglial modulation in the treatment of ischemic stroke and it provides additional information on microglial effector functions. This short review aims to elaborate on these TREM2-mediated microglial functions in the pathobiology and resolving of ischemic stroke.

## Introduction

Worldwide, stroke, of which ischemic stroke is the most prominent, is the second most common cause of death and is a major cause of permanent disability (1). Ischemic stroke is characterized by the loss of brain tissue and neuronal dysfunction due to a reduced blood flow to the affected brain areas. Microglia, the resident phagocytes of the central nervous system (CNS), continuously monitor the status of the brain parenchyma through extension and retraction of their processes (2). Furthermore, they are the first cells to become activated after stroke which migrate toward the site of injury to act as a first line of immune defense. Stroke outcome is determined by a fine balance of tissue-damaging pro-inflammatory and recovery-promoting pro-regenerative responses. Microglia play an important role in this inflammatory balance by obtaining a broadly defined disease promoting pro-inflammatory- or pro-regenerative phenotype (3–7). It should be noted that this classification will be used in the context of this review of ischemic stroke. There is an ongoing debate on phagocyte classification based on their activation state, activation stimuli, polarization, cellular origin, and phenotypical shift. However, this discussion will be left out of consideration but is thoroughly discussed by Ma et al. (7), Guilliams et al. (8), and Murray et al. (9).

Pro-inflammatory microglia dominate during the acute phase after stroke and are characterized by increased tumor necrosis factor alfa (TNF-α), interleukin (IL)-1β, IL-12, and inducible nitric oxide synthase (iNOS) expression amongst others. Pro-regenerative microglia (characterized by i.e., CD206 and Arginase-1 expression) play a protective role by stimulating tissue and vascular remodeling after stroke during the subacute and chronic phase. Pro-regenerative microglia secrete anti-inflammatory cytokines such as IL-4, IL-10, and transforming growth factor β (TGF-β) and remodeling factors including vascular endothelial growth factor (VEGF) and brain-derived neurotrophic factor (BDNF) (7, 10, 11). Unfortunately, these microglia numbers decline following an initial peak 12–24 h after stroke, whereas the number of pro-inflammatory microglia continue to rise, contributing to tissue damage and neuroinflammation (3, 10). Nonetheless, microglial polarization and the secretion of pro- and anti-inflammatory soluble factors remains a topic of interest in stroke research (10).

The clearance of apoptotic neurons and/or neuronal debris is an aspect of stroke repair that is often overlooked. Phagocytic clearance is indispensable in enabling the reconstruction and reorganization of neural networks and triggering tissue repair (12, 13). Moreover, in contrast to neuronal debris, phagocytosis of apoptotic cells is a controlled process that does not induce inflammation, thereby circumventing self-catalyzing secondary tissue damage that is characterized by free radical and nitric oxide production, glutamate excitotoxicity and the release of pro-inflammatory factors (14, 15). Recently, triggering receptor expressed on myeloid cells 2 (TREM2) emerged as a novel microglial target in stroke that is involved in phagocytosis and microglial function. TREM2 is expressed on myeloid cells including microglia in the CNS where its expression is over 400-fold higher than on other glial cells and neurons (16). The importance of this receptor in myeloid and microglial function is stressed by Nasu-Hakola disease, also known as polycystic lipomembranous osteodysplasia with sclerosing leukoencephalopathy. This disease develops in patients with a deleterious or loss of function mutation in the TREM2 gene, or in its signaling adapter protein DNAX activating protein of 12 kDa (DAP12) (17, 18). These patients develop bone cysts due to dysfunctional osteoclasts (19) but also present with progressive dementia, motor dysfunction, seizures and shortened life span (18, 20). Moreover, the R47H TREM2 mutation increases the risk for Alzheimer's disease (AD) 3–4 fold (21, 22) and mutations in the TREM2 gene are thought to be linked with the impaired uptake of Aβ-lipoprotein complexes by microglia (23).

The significance of TREM2 in ischemic stroke was shown by Kawabori et al. who showed that TREM2 deficiency was detrimental for ischemic injury and microglial phagocytosis (24). Moreover, TREM2 overexpression suppressed the pro-inflammatory reaction of microglia *in vitro* (25, 26). More strikingly, a recent study by Kurisu et al. demonstrated that specifically microglial TREM2 expression is fundamental in stroke outcome and not TREM2 expression on circulating macrophages (27). In addition to microglia, perivascular and perimeningeal macrophages can be seen as a distinct brain-resident macrophage with several indications for CNS diseases [reviewed in Faraco et al. (28)] which role has also recently emerged in stroke pathogenesis regulating vascular permeability and leukocyte chemotaxis in acute stroke (29). However, no reports are available describing the role of TREM2 in these cells and will therefore be left out of consideration. This review aims to provide an overview on the regulation and potential role of TREM2 in stroke pathobiology and resolution with a focus on microglial function as its role in these processes remains to be elucidated.

## Stroke Phagocytosis and Inflammation

The accurate clearance of apoptotic neurons and neuronal debris is crucial for the resolution of CNS damage. Following (ischemic) injury, microglia are activated, migrate toward the lesion site, secrete pro- and anti-inflammatory factors and clear cellular debris and apoptotic cells (13, 30). In general, proper clearance of apoptotic cells is mediated through four essential processes. The first step is initiated by apoptotic cells that release “find-me” signals to attract phagocytes to the site of neuronal injury. These signals include the nucleotides (2, 31) adenosine triphosphate (ATP) and uridine triphosphate (UTP), lysophosphatidylcholine (LPC) (32), sphingosine-1-phosphate (S1P) (33), and fractalkine/CX3CL1 (34). The second step involves recognition of the dying cells by “eat me” signals of which phosphatidylserine is the most prominent and well-studied (35). After recognition, the target cell becomes internalized and the engulfed target becomes degraded in the maturating phagosome (36). Interestingly, in the fourth phase, the phagocyte releases anti-inflammatory mediators such as TGF-β1, IGF-1, and IL-10 (37–39) and suppresses the production of pro-inflammatory factors (40). For in-depth information on phagocytosis and the associated cellular and molecular mechanisms, see reviews by Medina and Ravichandran (41), Park and Kim (42).

These phagocytosis mechanisms hold when injury is limited and these “find me” signals are present at relatively low concentrations. However, in ischemic stroke, extracellular ATP levels increase dramatically due to neuronal and glial depolarization waves and membrane leakage (14). These high levels of ATP then not only serve as a chemoattractant for microglia, but can activate P2 ×7 receptors which leads to the production of pro-inflammatory factors (43). In the subsequent phase of ischemic cell death, the dying neuronal cells leak intracellular signal molecules that together with extracellular molecules can be seen as danger signals that activate the immune system (44). These so-called danger associated molecular pattern molecules (DAMPs) include high concentrations of extracellular ATP (45), extracellular matrix breakdown proteins (46), heat-shock proteins (Hsp) (47), and the high mobility group box 1 protein (48). These DAMPs activate purine receptors and scavenger- or pattern recognition receptors on microglia, leading to the production of pro-inflammatory mediators by resident brain cells and infiltrating leukocytes (49). In this context, one of the most studied pattern recognition receptors is the membrane–bound Toll-like receptor (TLR) family in which the TLR2 and TLR4 subtypes are known to play an important role in ischemic stroke. TLR2 is most prominently expressed on microglia and TLR2 signaling increases the expression of pro-inflammatory and pro-apoptotic genes in the transient middle cerebral artery occlusion (tMCAO) mouse stroke model. Moreover, TLR2 deficient mice have a smaller infarct volume compared to controls (50). Similarly, TLR4-deficient mice had lower infarct volumes in a mouse model with permanent distal middle cerebral artery occlusion (dMCAO). Additionally, TLR4-deficient mice had a better score on a neurological and behavioral test. The lack of TLR4 decreased the expression of iNOS, cyclooxygenase-2 and matrix metalloprotease-9 amongst others, demonstrating an attenuation of the pro-inflammatory milieu that is created post-stroke (51).

Ultimately, acute stroke-associated inflammation is a self-limiting process that is coordinated by lipid mediators (52) that contribute to the effective removal of dead cells and debris, the generation of pro-regenerative milieu and the production of growth factors such as microglial IGF-1, VEGF, and BDNF that favor tissue regeneration. As will be discussed in the next section, TREM2 can interfere in the stroke immune response at multiple levels to determine stroke outcome.

## The Neuroimmunological Function of TREM2—Implications for Stroke Pathobiology

Microglia are key regulators of innate immunity in the CNS, protecting the parenchymal cells from injury. In addition to detecting extrinsic insults such as bacterial lipopolysaccharide (LPS), microglia are equipped with intrinsic DAMP-recognizing receptors like TLRs, as discussed previously. Similar to TLRs, TREM2 was shown to be capable of binding LPS and intrinsic ligands that are released during neuronal degeneration including lipids (53, 54), nucleic acids (24), and Hsps (55). However, the identification of the entire spectrum of endogenous TREM2 ligands and clarification of the ligand-specific physiological TREM2-mediated response in health and disease remains challenging [reviewed by Kober and Brett (56)]. Interestingly, fractalkine/CX3CL1-expressing damaged neurons stimulate TREM2 expression and attenuate neuronal excitotoxicity by enhancing the clearance of these injured neuronal cells by CX3CR1-expressing microglia (57, 58).

TREM2 is a single-pass transmembrane receptor that belongs to the immunoglobulin-like superfamily of receptors. The immunoglobulin-like ligand-binding domain is situated in the extracellular domain whereas the transmembrane domain facilitates the interaction with DAP12. This leads to the phosphorylation of the immunoreceptor tyrosine activation motif (ITAM) in DAP12 by Src kinases which subsequently activate Syk kinase to trigger intracellular signaling cascades involving the downstream signaling molecules phosphatidylinositol 3-kinase (PI3K), extracellular signal-regulated protein kinase (ERK), phospholipase Cγ (PLCγ), and Vav that influence microglial activation, function, survival, and phagocytosis (53, 54, 59–61). Interestingly, TREM2 can be shed from cell membranes by proteolytic cleavage by for example the sheddase ADAM17 (a disintegrin and metalloproteinase domain containing protein 17), forming soluble TREM2 (sTREM2) that is able to cross to the cerebrospinal fluid (62, 63). The current knowledge on the role of sTREM2 in CNS pathology and microglial function is mainly based on AD research. A recent paper by Zhong et al. demonstrated that the stereotactic injection of sTREM2 or adeno-associated virus mediated activation of sTREM2 reduced the amyloid plaque load and reduced functional memory deficits. Moreover, sTREM2 stimulated microglial proliferation and homing toward amyloid plaques where amyloid-β uptake and degradation was increased. Interestingly, these effects were specifically mediated by microglia as they were absent upon microglial depletion (64). Remarkably, sTREM2 levels in the cerebrospinal fluid were recently proposed as a biomarker for AD and the associated inflammatory response (63, 65, 66). Unfortunately, the role of sTREM2 in ischemic stroke is to date unexplored, and will therefore be left out of consideration for stroke specific microglial TREM2 function in this context.

**Figure 1:**
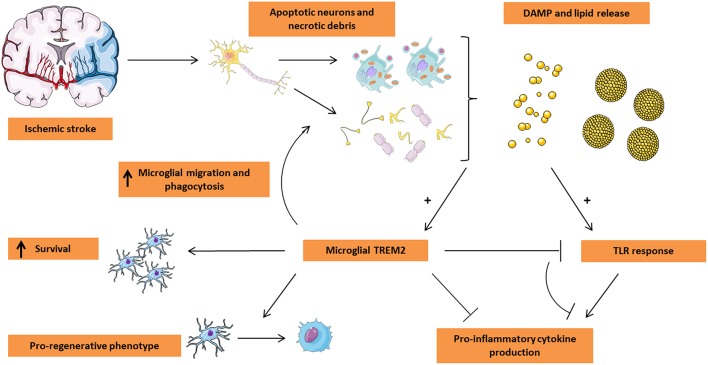
Microglial TREM2 in stroke pathogenesis. Ischemic stroke leads to the massive loss of neuronal tissue by triggering apoptosis and/or necrosis. In turn, the dying tissue releases damage-associated molecular pattern (DAMP) molecules including nucleotides and ECM breakdown products in addition to lipid mediators being present in myelin and neuronal debris and on the surface of apoptotic cells. These events trigger both TLR and TREM2 activation on microglia. While classically the TLR response leads to a pro-inflammatory microglial phenotype characterized by the release of pro-inflammatory cytokines, TREM2 activation appears to counteract the TLR response, leading to a decrease in the production of pro-inflammatory cytokines. Moreover, microglial TREM2 stimulates migration of microglia toward the lesion site and promotes the phagocytic clearance of apoptotic cells and debris. Finally, TREM2 was shown to be associated with a pro-regenerative microglial phenotype and the production of pro-regenerative cytokines and is suspected to play a role in microglial survival. Based on these functions of TREM2, this receptor is an attractive target for microglial modulation in the treatment of ischemic stroke. This Figure was created using Servier Medical Art licensed under a Creative Common Attribution 3.0 Generic License, available online at http://smart.servier.com/.

Based on these TREM2 effects on microglial function and the role of microglia in ischemic stroke, several mechanisms are proposed to be mediated by microglial TREM2 activity (Figure 1). The first and potentially the most thorough investigated role of TREM2 in stroke is its function in phagocytosis. A pioneer study by Takahashi et al. (59) demonstrated that microglial TREM2 is involved in the phagocytosis of apoptotic neurons. In this study, knockdown of TREM2 with short hairpin TREM2 RNA (shRNA) in primary mouse microglia decreased the phagocytic clearance of apoptotic neurons which coincided with the increased expression of pro-inflammatory TNF-α and nitric oxide synthase-2. Lentiviral overexpression and activation of TREM2 increased phagocytosis and attenuated the pro-inflammatory response. In addition to its role in phagocytosis, Takahashi et al. showed that the migratory capacity of microglia with activated TREM2 toward CCL21 is increased significantly. CCL21 is used by ischemic injured neurons as a “find me” signal to attract microglia, suggesting a role of TREM2 in the early phase after ischemic injury (67). Kawabori et al. demonstrated that TREM2 deficiency exacerbates ischemic injury in a dMCAO mouse stroke model in which TREM2 knockout (KO) and wildtype (WT) mice were compared (24). *In vitro*, knockdown of TREM2 in the BV-2 mouse microglial cell line using small interfering RNA (siRNA) directed against TREM2 inhibited phagocytosis of Neuro-2A cells that were rendered apoptotic by oxygen- and glucose deprivation (OGD), an *in vitro* model that mimics ischemic damage. This role of TREM2 in phagocytosis was also demonstrated *in vivo*. TREM2 deficient mice showed a reduced tissue resorption and therefore increased infarct size following ischemic damage compared to WT controls. Confocal microscopy confirmed these results as a significantly reduced direct contact between activated macrophages and TUNEL-positive dying cells at the stroke site in TREM2 KO mice was observed (<4% compared to almost 40% in WT animals). The reduced phagocytic clearance of apoptotic cells and debris in TREM KO mice was also reflected by the virtual absence of Oil Red O-stained foamy macrophages which stained positive due to the uptake of lipid-containing cellular debris and dead cells. In line with these histological findings, the Bederson score and ladder test, which both measure neurological deficits, demonstrated a worsened functional outcome in the TREM2 KO mice. Moreover, WT mice showed a gradual improvement in the ladder test to nearly baseline levels while TREM2 KO mice did not reach these values over 2 weeks (24). This stresses the importance of clearing neuronal debris to enable synaptic and axonal repair, as stated previously (13). More recently, Kurisu et al. demonstrated that it is specifically microglial TREM2 that is essential in stroke outcome by using bone marrow chimeric mice in which TREM2 KO mice were used as donor and WT mice as recipient. The results of this study indicated improved neurological outcome in the elevated body swing and adhesive removal test and smaller infarct volume in mice with intact TREM2. In addition, mice with no intact microglial TREM2 demonstrated a lower number of phagocytes near the stroke lesion that also showed reduced phagocytic activity (27). Similar observations have been made in the experimental autoimmune encephalomyelitis (EAE) model of multiple sclerosis (68) and in the cuprizone-induced non-autoimmune demyelination model (54, 69). In EAE, TREM2 overexpressing bone-marrow derived macrophages demonstrated enhanced phagocytosis of apoptotic neurons and after intravenous injection, these cells cleared myelin debris and ameliorated the disease outcome (68). Supporting the role of TREM2 in EAE, Piccio et al. showed that inhibiting TREM2 activation is detrimental for EAE outcome (70). In the cuprizone model, the corpus callosum in TREM2 KO mice showed extensive demyelination, axonal damage and decreased clearance of myelin debris compared to WT controls (54, 69). Furthermore, Poliani et al. showed that myelin-associated lipids triggered microglial TREM2 signaling, stressing the need of microglial TREM2 for effective clearance of myelin debris (54). More support for the importance of microglial TREM2 in the phagocytic clearance of apoptotic cells, comes from AD research. Atagi et al. demonstrated that apoptotic neuronal cells have the ability to bind Apolipoprotein E (APOE), the major cholesterol carrier protein in the brain. APOE was demonstrated to be a TREM2 ligand that enhanced the microglial phagocytosis of apoptotic neurons and the phagocytic capacity of microglia was diminished in the AD-associated TREM2-R47H mutant (71). While ongoing research is focusing on the link in AD between TREM2 mutants and the APOEε4 isoform, which is the major risk factor for AD, the association of TREM2 and APOE in ischemic stroke and their putative intertwined signaling remains unclear. However, given the ability of apoptotic neurons to bind APOE and use it as an opsonisation mechanism to enhance TREM2-mediated microglial phagocytosis demonstrates that this ligand-receptor interaction plays in important role in the clearance of damaged neurons (72).

Second, TREM2 can drive the pro-inflammatory stroke microenvironment toward a pro-regenerative environment and can therefore exert a neuroprotective effect on the ischemic brain. In a study by Wu et al., TREM2 expression is upregulated in microglia following OGD *in vitro*, and in the ischemic penumbra after tMCAO in mice (25). *In vivo*, TREM2 silencing exacerbated stroke outcome as demonstrated by an increased lesion volume, the number of apoptotic neurons and a decrease in neurological function measured with the Modified Garcia Score. This correlated with an increase in TNF-α, IL-1β, and iNOS mRNA levels *in vitro* in microglia exposed to OGD. Conversely, TREM2 overexpression reduced TNF-α, IL-1β, and iNOS mRNA levels and decreased neuronal apoptosis *in vitro*. Moreover, this study demonstrated the role of the ERK and NF-κB in TREM2 signaling. TREM2 silencing increased phosphorylation of ERK and NF-κB in primary microglia exposed to OGD. Taken together, a tempering role of TREM2 signaling in pro-inflammatory pathways and a critical protective role for TREM2 in ischemia-induced neuronal injury is suggested (25). These findings are in line with Zhai et al. (26) that demonstrated that intraperitoneal administration of hsp60, a TREM2 agonist (55), or intraventricular injection of a TREM2 lentivirus had a neuroprotective effect *in vivo*. This study reported a decreased number of apoptotic cells and a smaller infarct volume. Interestingly, TREM2 expression is increased by an IL-4/IL-13 treatment, cytokines that induce a pro-regenerative phenotype in microglia whereas its expression is decreased after exposure to the pro-inflammatory polarizing factors LPS and interferon-γ. Therefore, the authors suggest TREM2 as a novel pro-regenerative microglial marker. Moreover, pro-inflammatory iNOS, TNF-α, and IL-6 expression was increased in OGD-exposed primary microglia and treated with TREM2 shRNA while Arginase-1 and BDNF levels were increased when these cells were treated with a TREM2 vector, indicating a pro-regenerative phenotype. In contrast to these studies and the research of Kawabori et al. that suggest a role of TREM2 in alleviating the pro-inflammatory response and its beneficial role in stroke outcome, Sieber et al. showed that in the subacute phase after stroke, the transcription of the pro-inflammatory cytokines IL-1α, IL-1β, and TNF-α was reduced in TREM2 KO mice (73). However, a difference in lesion size in WT and TREM2 KO mice was not observed. These opposing results may be explained from differences between the timing of the analysis, although with the exception of Zhai et al., peak TREM2 levels were observed 7 days post-ischemia. Given this 7 days period for TREM2 to reach peak levels and the abovementioned experiments, it can be postulated that a gradual increase in TREM2 expression is associated with a dampening of the pro-inflammatory microglial response elicited in the acute and sub-acute stroke phase. Another possibility for these opposing results is the stroke model used, although only Kawabori et al. used the dMCAO model compared to the filament model used by Zhai et al., Wu et al., and Sieber et al. Despite these different results, the majority of these stroke studies suggest a beneficial role of TREM2 in ischemic stroke (24–26). It should be noted that to date, no stroke stage-specific reports are available that go into detail in TREM2 expression dynamics and pathways that lead to TREM2 upregulation and effector mechanisms. This is of specific importance as TREM2 is a microglia-specific receptor involved in stroke resolution and microglial function varies between the acute, sub-acute and chronic stroke phase balancing between a pro-inflammatory and pro-regenerative phenotype (6, 7).

A third indication of the role of TREM2 in stroke is that TREM2 can inhibit the TLR-mediated pro-inflammatory response. This was first demonstrated in myeloid cells by Turnbull et al. who showed that in macrophages isolated from WT and TREM2 KO mice, the production of pro-inflammatory mediators was elevated in TREM2 KO macrophages after LPS stimulation (74). Similarly, Hamerman et al. showed an increased TLR-LPS response in DAP12 deficient mice and demonstrated that TLR responses are negatively regulated by TREM2 and DAP12 (75, 76). Although these studies show a connection between the TLR response and TREM2 signaling, mechanistic insights in the TLR-TREM2 interaction come from a study by Peng et al. using bone-marrow derived macrophages (77). The ERK pathway can be activated by TLR4 binding of LPS leading to the production of pro-inflammatory cytokines (78, 79). In the study by Peng et al., it was shown that the adapter protein Dok3 is phosphorylated by DAP12-ITAM which subsequently inhibits the RAS-ERK pathway, thereby reducing the production of pro-inflammatory cytokines such as IL-6 and TNF-α (77). However, the role of the TREM2-TLR interaction in ischemic stroke remains to studied into more detail. A recent study by Rosciszewski et al. (80) investigated this interaction on astrocytes, although it should be noted that TREM2 expression in microglia is almost 400 times higher than in astrocytes (16). This study showed that DAP12 overexpression suppressed LPS-induced astrocyte NF-κB activation *in vitro*, diminishing the pro-inflammatory response. Moreover, TREM2 expression in astrocytes was observed up to 14 days post-lesion in the ischemic penumbra. These results suggest that TREM2 expression in astrocytes negatively regulates the TLR4-mediated pro-inflammatory response by reducing NF-κB activation (80).

Finally, TREM2 signaling is associated with microglial survival. Sieber et al., Kurisu et al., and Kawabori et al. observed a decreased number of (activated) microglia in TREM2 KO models compared to WT controls (24, 27, 73). However, Kurisu et al. demonstrated that the number of microglia in the non-ischemic brain is similar between TREM2 KO and WT mice (27). Therefore, the diminished number of microglia associated with the stroke lesion in TREM2 KO mice is due to the reduced chemotactic capacity in KO mice, which is supported by a previous study that reports a reduced microglial recruitment to sites of laser-induced neuronal injury in TREM2 KO mice (81). These results were in line with the cuprizone model used by Poliani et al. and Cantoni et al. who also showed decreased microglia numbers in TREM2 KO mice (54, 69). However, no plausible mechanism is identified. Nonetheless, the role of TREM2 in microglial survival and proliferation cannot be excluded, as Zheng et al. reported a reduction in microglial proliferation rate after TREM2 downregulation (82). However, no analysis was performed to unravel the TREM2 targets involved with the decreased proliferation rate. Other reports on the involvement of TREM2 in microglial proliferation come from AD research. A possible mechanism of TREM2-sustained microglial survival come from Wang et al. who demonstrated in an AD model using TREM2 KO 5XFAD mice that microglial survival is diminished compared to 5XFAD control mice. They determined that TREM2 signaling is essential for microglial survival at low concentrations of colony-stimulating factor 1 (CSF-1). The same group showed that TREM2 synergizes with CSF-1-CSF-1 receptor signaling, contributing to microglial survival. Moreover, this study suggests that the lipid-sensing function of TREM2 provides a survival function in addition to triggering phagocytosis of apoptotic cells and myelin debris (53).

## Concluding Remarks

Microglia play an essential role in both the pro-inflammatory and pro-regenerative response that is triggered following ischemic injury and can therefore be seen as a double-edged sword in stroke pathobiology. TREM2 signaling mediates microglial phagocytosis of myelin debris and apoptotic cells, microglial migration and survival, and can drive the microglial activation status toward an anti-inflammatory phenotype. Therefore, targeting TREM2 signaling has become a therapeutic target as it was shown that the systemic administration of a TREM2 agonist or TREM2 overexpression had a neuroprotective effect in ischemic injury. However, it should be noted that the temporal dynamics of TREM2 expression and effector mechanisms in ischemic stroke remain to be elucidated and current insights in TREM2 function are mainly derived from different disease models and cell types. Nonetheless, the role of TREM2 in ischemic stroke is mainly considered beneficial, and the same holds for its role in AD and demyelinating disease although few studies report a detrimental effect of TREM2 signaling in these neural diseases (83). Moreover, the effect of sTREM2 in ischemic stroke remains unexplored to date and given its implications in AD pathobiology, sTREM2-signaling provides an attractive new target in the stroke research field. Despite the great promise for TREM2 in ischemic stroke, TREM2 signaling has opposite effects in peripheral nerve injury including motor nerve injury (84) and neuropathic pain (85), and after traumatic brain injury (86). This stresses the importance of additional disease-specific understanding of the TREM2-DAP12 signaling effects. Nonetheless TREM2 provides an attractive target for microglial modulation in the treatment of ischemic stroke and it offers additional information on microglial effector functions in this devastating disease.

## Author Contributions

PG conceived and wrote the manuscript. IL revised and edited the manuscript. All authors listed have made a substantial, direct, and intellectual contribution to the work, and approved it for publication.

### Conflict of Interest Statement

The authors declare that the research was conducted in the absence of any commercial or financial relationships that could be construed as a potential conflict of interest.
